# Lyme disease risk in southern California: abiotic and environmental drivers of *Ixodes pacificus* (Acari: Ixodidae) density and infection prevalence with *Borrelia burgdorferi*

**DOI:** 10.1186/s13071-016-1938-y

**Published:** 2017-01-05

**Authors:** Andrew J. MacDonald, David W. Hyon, John B. Brewington, Kerry E. O’Connor, Andrea Swei, Cheryl J. Briggs

**Affiliations:** 1 Department of Ecology, Evolution and Marine Biology, University of California, Santa Barbara, CA 93106-9620 USA; 2 Department of Biology, Stanford University, 371 Serra Mall, Stanford, CA 94305 USA; 3 Department of Biology, San Francisco State University, 1600 Holloway Avenue, San Francisco, CA 94132 USA

**Keywords:** Partial least squares regression, *Ixodes spinipalpis*, *Ixodes peromysci*, *Borrelia bissettiae*, Enzootic transmission, Vector diversity, Drought

## Abstract

**Background:**

Tick-borne diseases, particularly Lyme disease, are emerging across the northern hemisphere. In order to manage emerging diseases and predict where emergence will likely occur, it is necessary to understand the factors influencing the distribution, abundance and infection prevalence of vector species. In North America, Lyme disease is the most common vector-borne disease and is transmitted by blacklegged ticks. This study aimed to explore the abiotic and environmental drivers of density and infection prevalence of western blacklegged ticks (*Ixodes pacificus*) in southern California, an understudied and densely populated region of North America.

**Results:**

Over the course of this two-year study, densities of *I. pacificus* adults were consistently positively associated with host availability for juvenile ticks and dense oak woodland habitat. Densities of nymphal and larval *I. pacificus*, on the other hand were primarily predicted by host availability for juvenile ticks in the first year of the study, and by habitat characteristics such as dense leaf litter in the second year. Infection with the causative agent of Lyme disease, *Borrelia burgdorferi* (*sensu stricto*), and related spirochetes was not predicted by the abiotic conditions promoting *I. pacificus* populations, but rather by diversity of the tick community, and in particular by the presence of two *Ixodes* tick species that do not generally feed on humans (*Ixodes spinipalpis* and *Ixodes peromysci*). *Borrelia* spp. infection was not detected in the *I. pacificus* populations sampled, but was detected in other vector species that may maintain enzootic transmission of the pathogen on the landscape.

**Conclusions:**

This study identified dense oak woodlands as high-risk habitats for *I. pacificus* tick encounter in southern California. The shift in relative importance of host availability to habitat characteristics in predicting juvenile tick abundance occurred as California’s historic drought intensified, suggesting that habitat providing suitable microclimates for tick survivorship became centrally important to patterns of abundance in the face of deleterious abiotic conditions. These results underscore the need for further investigation of the effects of climate change on tick-borne disease in California. Finally, despite low risk of human Lyme disease infection posed by *I. pacificus* in southern California, evidence of infection was found in other tick species, suggesting that enzootic transmission of tick-borne borreliae may be occurring in southern California, and involve parallel enzootic cycles with other tick and host species but not necessarily humans.

## Background

In recent decades numerous vector-borne zoonotic diseases (VBZDs) have emerged, including West Nile virus, chikungunya, Zika virus, Powassan virus and tick-borne relapsing fever. Further, endemic regions where VBZDs have historically circulated are experiencing increases in human incidence and transmission intensity in vector populations and wildlife hosts [1, 2]. Transmission of VBZDs to humans requires the interaction of human populations with natural pathogen transmission cycles between competent vectors and reservoir hosts at the human-animal interface [3]. Understanding the factors that influence the distribution and abundance of vector species [2], as well as infection prevalence in vector populations is thus critical to an understanding of human risk of infection with VBZDs. For VBZDs, because humans are often dead-end hosts and do not maintain pathogen transmission cycles, infection prevalence in vector populations and thus risk of transmission to humans are driven primarily by wildlife reservoirs and enzootic pathogen transmission cycles [2]. Management and control of such zoonoses is quite difficult because vaccination or treatment of human populations has no effect on underlying enzootic transmission, and infection is determined largely by contact with vectors [2, 4]. Strategies for disease control involving environmental management or land use planning [5, 6] may be increasingly important due to development of resistance to insecticides in vector populations and the challenges associated with managing enzootic transmission cycles [7, 8]. Such management strategies require an understanding of the abiotic and environmental conditions that promote both vector populations and elevated infection prevalence with zoonotic pathogens on the landscape.

In this study, the influence of environmental and abiotic factors on densities of the primary vector of Lyme disease in the western United States (US), *Ixodes pacificus*, as well as infection prevalence of vector ticks with the causative agent in North America, *Borrelia burgdorferi* (*sensu stricto*) (*s.s*.), were investigated in southern California. Tick-borne diseases, such as Lyme disease, are particularly challenging to manage because tick populations are difficult to control [8] and environmental or landscape risk factors conducive to high tick abundance are often not well understood [9]. Lyme disease, which is caused by the spirochete bacterium *B. burgdorferi* (*s.s*.) and vectored by ticks in the genus *Ixodes*, is one of the most common tick-borne diseases globally. In North America, there are distinct Lyme disease foci in the eastern, upper midwestern and far western regions of the US and Canada. In the far western US, human incidence of Lyme disease is highest in northwestern California and investigations of the ecology of this disease system have largely focused on that region. In northern California, dense oak woodland habitats tend to harbor the highest densities of *I. pacificus* ticks, particularly for the larval and nymphal stages; they also experience higher nymphal infection prevalence with *B. burgdorferi* (*s.s*.), both key elements of entomological risk of human infection [10–12]. Within oak woodland habitats, temperature - particularly maximum summer temperatures - relative humidity, elevation, aspect and presence of dense leaf litter have been found to be important predictors of tick density and/or infection prevalence [13–15]. These associations suggest that a combination of direct, e.g. accelerated molting and mortality due to high summer temperatures [13], and indirect, e.g. influences on densities of key tick hosts and pathogen reservoirs, effects of abiotic and habitat conditions determine tick density and infection prevalence in northwestern California [15].

However, there is evidence that suggests these abiotic and environmental factors may not be predictive of entomological risk in central and southern California where nymphal *I. pacificus* questing behavior appears to differ markedly from that observed in Lyme-endemic northwestern California [16, 17]. Densities of questing nymphal *I. pacificus* are extremely low in oak woodland sites in southern California [16, 17] relative to infestation of western fence lizards (*Sceloporus occidentalis*), a key host for juvenile *I. pacificus* and an effective sentinel animal for detecting juvenile tick activity in this region [17]. This suggests both that: (i) risk of human exposure to nymphal *I. pacificus* in southern California is relatively low and likely to be highly localized to areas with habitat types, abiotic conditions and host assemblages that promote nymphal questing activity; and (ii) due to comparatively high densities of questing adult *I. pacificus* relative to nymphs, possibly due to differences in questing behavior in the nymphal stage [16], exposure to adult female ticks may present greater risk in central and southern California than exposure to nymphal ticks [16, 17].

Thus, in this study we were interested in determining whether dense oak woodland habitats are associated with elevated densities of nymphal *I. pacificus* and whether different habitat and abiotic conditions might be better predictors of exposure to adult *I. pacificus*, and therefore aggregate entomological risk, in southern California. Given the low densities of nymphal *I. pacificus* observed in previous studies [16, 17], we expected to find elevated densities of nymphal ticks to be narrowly associated with dense oak woodland habitat, higher in elevation, with dense leaf litter in the understory, which together promote microclimatic conditions found to be important to nymphal tick abundance in northern California [10, 11, 14, 15]. Similarly, we expected elevated adult tick density to be associated with dense oak woodland, or forest edge habitats where deer, important reproductive hosts for adult ticks, tend to forage. Additionally, we addressed whether these same abiotic and habitat conditions predict vector tick infection with *B. burgdorferi* (*s.s*.) on the landscape or whether this pathogen may be associated with different habitats and environmental conditions in southern California. Again, we expected a similar pattern, with elevated infection prevalence associated with dense oak woodland habitats as in northern California [10, 14, 15]. To elucidate the habitat and abiotic factors associated with elevated entomological risk (e.g. elevated densities of infected ticks) for Lyme disease in this understudied region, tick surveys were conducted in Santa Barbara County, California. Over 2 years, surveys were conducted in plots that represent a range of habitats with varying environmental and abiotic conditions.

## Methods

### Field sites, tick sampling and environmental data collection

**Fig. 1:**
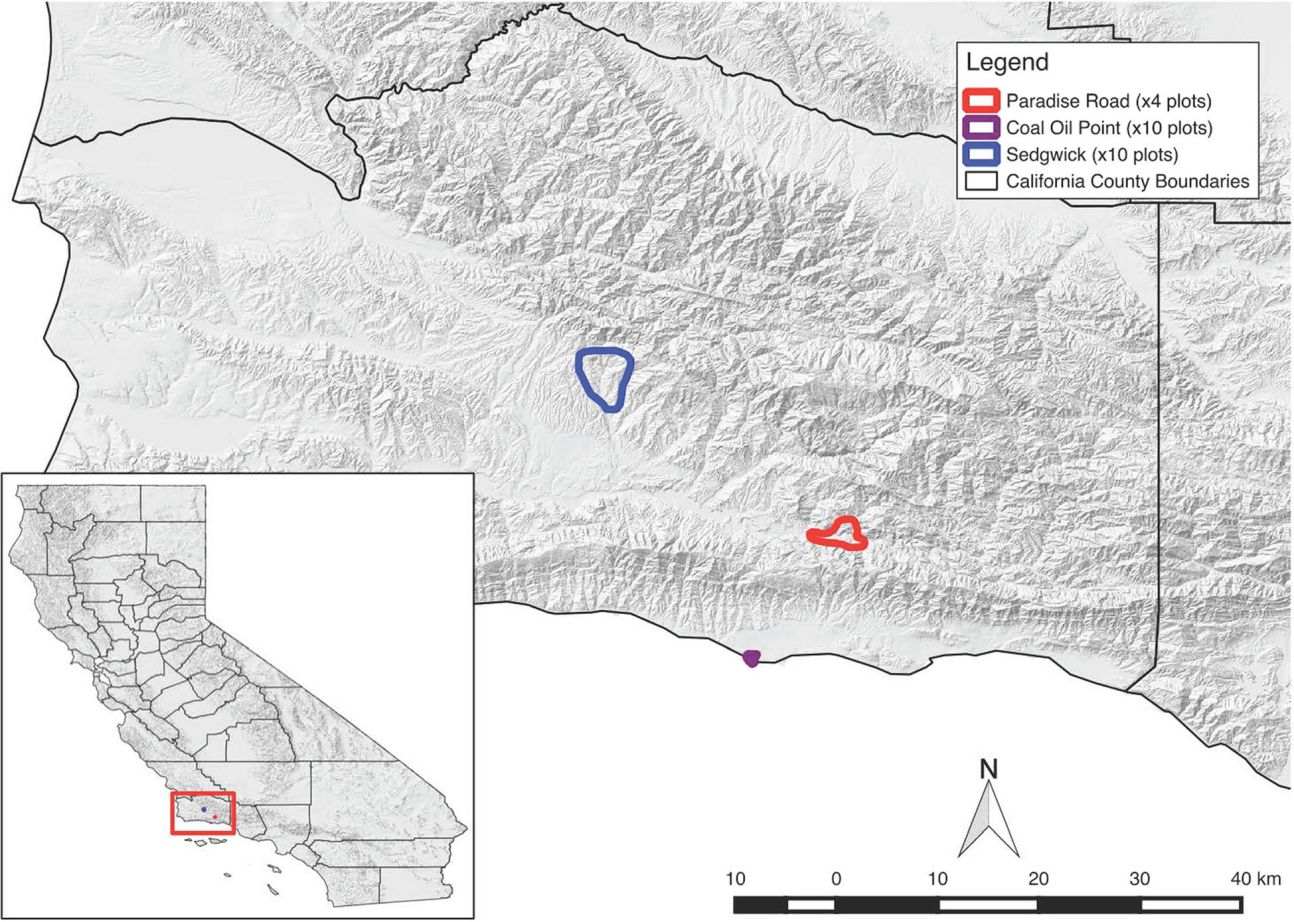
Map of study sites. State of California is inset on the *bottom left*; Santa Barbara County is in the main frame with study site boundaries included. California hill shade data layer was obtained from Cal-Atlas through https://koordinates.com (https://koordinates.com/layer/692-california-hillshade-30m/)

Questing ticks were collected in 24, 50 × 50 m plots across three sites in Santa Barbara County, California. Climatic conditions in this region are Mediterranean with cool, wet winters and warm, dry summers with microclimatic variation driven largely by topography and habitat characteristics. The three sites selected were: (i) Sedgwick Reserve (34°42'04.38"N, 120°02'50.81"W), a 2388 ha reserve that is part of the University of California Natural Reserve System (UCNRS) and located in the Santa Ynez Valley (×10 plots); (ii) Paradise Reserve (34°32'22.07"N, 119°47'51.89"W), a ~67 ha privately owned natural area located on the north side of the Santa Ynez Mountains in the Los Padres National Forest (× 4 plots); and (iii) Coal Oil Point Reserve (34°24'52.96"N, 119°52'48.59"W), a 69 ha coastal reserve that is part of the UCNRS and located west of the University of California, Santa Barbara campus (× 10 plots) (Fig. 1). These three sites were chosen because they represent a range of habitat types, abiotic conditions and degree of maritime influence common in this region. Coal Oil Point experiences a significant marine influence, which moderates temperature extremes and provides fog water subsidies, with habitat dominated by coastal scrub, grassland and patches of coast live oak (*Quercus agrifolia*). Sedgwick experiences less maritime influence, with generally warmer summers and colder winters, with habitats dominated by oak woodland on north facing slopes, oak savannah in valleys, as well as grassland and chaparral/scrub. Paradise Reserve is dominated by oak woodland with patches of open grassland and chaparral/scrub habitat interspersed, and experiences lower temperature extremes than Sedgwick, but less maritime influence than Coal Oil Point. Plots were chosen within each of these three sites using a stratified-random design to ensure that different habitat types (oak woodland, oak savannah and chaparral/grassland) with a range of abiotic and environmental conditions were sampled equally across all three sites. Habitat types were classified based on satellite imagery from Google Earth and subsequently ground-truthed (see below). Plots were chosen randomly, and located at least 200 m apart, within each broad habitat type with the aid of Quantum GIS, an open source Geographic Information System [18].

This study was conducted from 2013 to 2015, during which time California was experiencing drought conditions (beginning 2011 through 2015). Data on plot-specific habitat, abiotic and environmental variables were collected each year of the study, and chosen based on previous studies [10, 13, 15]. Data loggers, placed in each plot just above ground level and protected from direct solar radiation, collected hourly temperature data during both summer and winter months (iButtons, Maxim Integrated, San Jose CA). From the data loggers, we calculated average maximum and minimum daily temperature over the dry (1 May - 31 October) and rainy (1 November-30 April) seasons. We estimated overstory canopy cover in each plot using satellite imagery derived from Google Earth and processed in Quantum GIS [18], which we subsequently ground-truthed with a convex spherical crown densitometer (Forestry Suppliers, Inc. Jackson MS). We estimated percent cover of leaf litter greater than 5 cm in depth, grass/herbaceous vegetation, understory woody vegetation (e.g. *Artemisia californica*, *Baccharis pilularis*, *Toxicodendron diversilobum*) and bare ground following the same procedure as above. Additionally, we measured stem density (number of stems greater than 5 cm in diameter at breast height and greater than 1.5 m in height), slope and elevation at the center of each plot, as well as density of inhabited dusky-footed woodrat (*Neotoma fuscipes*) middens [19]. While other species of small vertebrate host are known to be important blood meal hosts for juvenile ticks (e.g. western fence lizards, *Sceloporus occidentalis*) and pathogen reservoirs (e.g. western gray squirrels, *Sciurus griseus*), these species were not censused in this study. Instead, woodrat middens were censused because *N. fuscipes* is an important reservoir for *B. burgdorferi* (*s.s*.) in California, the nests are conspicuous on the landscape and the density of active nests may be indicative of small vertebrate host availability for the immature stages of *I. pacificus* more generally in this region [20, 21]. Density of inhabited woodrat middens was estimated once each year for each of the 24 plots by counting every active nest within each plot during the spring (March-April) of each year of the study, following Hamm and colleagues [19]. Measurements of relative humidity, while thought to be an important driver of tick survivorship during the summer dry season [13, 14], were not collected at the plot-level. However, weather station data from the region indicate that high summer- and winter-time temperatures are negatively correlated with measures of relative humidity [16], suggesting that plot-level temperature and habitat characteristics alone are a good proxy for this metric of microclimatic conditions.

Questing ticks were collected at each site using the flagging method, in which a 1 m^2^ white flannel cloth is dragged over understory vegetation and leaf litter, and attached, questing ticks are removed (e.g. [22]). This method is effective for collection of ixodid ticks and provides a proxy for human risk of tick encounter. An area of 500 m^2^ was sampled on each sampling event in each of the 24 plots, which were sampled weekly to bi-weekly from late November to early June, the period of seasonal activity for *I. pacificus* in southern California [16] in order to determine both peak and average density of each life stage of *I. pacificus* in each plot. All 24 plots across the three reserves were sampled during the 2013–2014 and 2014–2015 seasons. All ticks encountered were collected and preserved in 70% ethanol for species identification and subsequent testing in the lab for infection with *B. burgdorferi* (*sensu lato*) (*s.l*.), the genospecies complex containing the causative agent of Lyme disease.

### DNA extraction and pathogen detection

DNA was extracted from all adult and nymphal *Ixodes* spp. ticks using a DNeasy blood and tissue kit (Qiagen, Valencia, CA) following the manufacturer’s instructions. All tick samples were then screened for infection with spirochetes in the *B. burgdorferi* (*sensu lato*) (*s.l*.) complex, of which *B. burgdorferi* (*s.s*.) is a part, *via* nested polymerase chain reaction (PCR) targeting the 5S-23S rRNA spacer region of all borreliae belonging to this group, following the methods outlined in Lane et al. [23]. PCR-positive samples were sequenced at the 5S-23S intergenic spacer region following Lane et al. [23], on an ABI Prism 3100 Genetic Analyzer (Applied Biosystems, CA).

While *I. pacificus* is the primary vector of *B. burgdorferi* (*s.s*.) to humans, other species of ticks in the genus *Ixodes* have been implicated in enzootic transmission of various genospecies within the *B. burgdorferi* (*s.l*.) complex [24, 25]. Given the relative dearth of previous studies on Lyme disease ecology in central and southern California, and the low prevalence in vector populations from these few investigations [17, 26], DNA from all adult and nymphal *Ixodes* spp. ticks was extracted and tested individually for infection with *B. burgdorferi* (*s.l*.) complex spirochetes.

### Statistical analyses

Habitat and abiotic predictor variables, which were selected *a priori* based on previous studies [10, 13, 15], were highly collinear in this study, and a comparatively large number of predictor variables of interest were measured relative to the number of observations (i.e. 10 to 13 predictor variables, depending on the model, and 24 sampled plots). To address the problem of multicollinearity and sample size, data dimensionality reduction was necessary in the statistical analyses employed. Partial least squares regression (PLSR) is particularly well suited to addressing these problems and has been shown to perform better than multiple regression or principal components regression techniques in similar ecological data analysis contexts [27].

PLSR generalizes and combines features of principal component analysis and multiple regression to (i) eliminate the problem of multicollinearity in the independent variables (***X***) that plagues the ordinary multiple regression approach, and (ii) eliminate the problem of choosing an *optimum* subset of predictors that remains in the principal components regression approach. In principal components regression, orthogonal components are chosen that explain as much of the *variance* in ***X*** as possible, which does not guarantee that the components chosen are relevant to the dependent variable of interest (***Y***) when subsequently used as predictors in a regression framework. In contrast, in PLSR a set of components, or *latent vectors*, are chosen that explain as much of the *covariance* between ***X*** and ***Y*** as possible. This is followed, as in principal components regression, by a regression step in which the set of orthogonal components chosen through the simultaneous decomposition of ***X*** and ***Y*** are used to predict ***Y***.

PLSR models were specified using the abiotic and environmental data described above as predictors, and both peak and average density of adult, nymphal and larval *I. pacificus* ticks as well as infection prevalence (proportion infected) of *Ixodes* spp. ticks with *B. burgdorferi* (*s.l*.) as outcome variables. Environmental data measured in the year prior to tick collection, in addition to data from the year of collection, were included in the models because abundance and infection prevalence of adult and nymphal ticks in year *t* are largely determined by survivorship and activity of nymphal and larval ticks in year *t* – 1, due to the 3-year life-cycle of *I. pacificus* [13]. Concurrent temperature and relative humidity may also influence tick questing activity [13, 14], so average daily maximum rainy season temperature from the year of collection was also included in all models. Models specified for the first year of the study include only concurrent rainy season temperature, and no measure of lagged summer or winter temperature, because data were not available for the previous year. Models were specified independently for the different years of the study to both investigate the robustness of the core results, as well as to separate natural interannual variation in tick density that might be influenced by variation in environmental conditions from year to year. All statistical analyses were conducted in R 3.2.4 [28], and PLSR models were run using the package ‘*plsdepot*’ [29].

## Results

### Drivers of tick density

In total, 765 *I. pacificus* ticks - 288 adults, 67 nymphs and 410 larvae - were collected over the duration of the study across all 24 plots. In addition, 9 *Ixodes spinipalpis*, 6 *Ixodes peromysci*, 178 *Ixodes brunneus*, 257 *Haemaphysalis leporispalustris* (rabbit tick), 544 *Dermacentor variabilis* (American dog tick), and 525 *Dermacentor occidentalis* (Pacific Coast tick) were collected over the course of the study (Table 1). The large number of *I. brunneus*, a parasite of migratory and other birds, collected in this study was likely due to the high density and diversity of bird species documented at Coal Oil Point Reserve (http://coaloilpoint.ucnrs.org/Species/Birds.html) where the majority of the *I. brunneus* were collected. Both average and peak density of adult and nymphal *I. pacificus* declined slightly from the 2013–2014 to the 2014–2015 season, though not significantly (Table 2). In contrast, average and peak larval *I. pacificus* density increased slightly, though not significantly, from 2013–2014 to the 2014–2015 seasons (Table 2). Separate PLSR models were constructed for each tick response variable for each year of the study. Results were remarkably consistent between models predicting peak and average density of adult, nymphal and larval *I. pacificus* within a given year, due to significant positive correlation between these two measures, so multivariate PLSR models were run with both peak and average tick density as outcome variables in the same model. The results of the multivariate models of tick density, as well as the models predicting infection prevalence are presented here.

**Table 1:** Tick species collected by flagging by stage, year and site. Tick species diversity (Shannon’s H index) is presented as maximum plot-level diversity with average reserve-level diversity in parentheses

Site	Year	*I. pacificus* (A, N, L)	*I. brunneus* (A, N, L)	*I. spinipalpis* (A, N, L)	*I. peromysci* (A, N, L)	*D. occidentalis* (A, N, L)	*D. variabilis* (A, N, L)	*H. leporispalustris* (A, N, L)	Tick diversity maximum (average)
COPR	2014	31, 0, 0	1, 1, 20	0, 3, 0	0, 1, 0	0, 0, 0	390, 0, 0	1, 29, 151	1.365 (0.315)
	2015	16, 0, 0	0, 3, 152	1, 5, 0	1, 4, 0	0, 0, 0	125, 0, 0	0, 8, 33	
Paradise	2014	93, 32, 89	0, 0, 0	0, 0, 0	0, 0, 0	12, 1, 8	0, 0, 0	0, 0, 0	0.414 (0.329)
	2015	61, 1, 35	0, 0, 0	0, 0, 0	0, 0, 0	17, 0, 0	0, 0, 0	0, 0, 0	
Sedgwick	2014	54, 8, 35	0, 0, 0	0, 0, 0	0, 0, 0	246, 15, 114	24, 0, 5	0, 7, 24	0.982 (0.613)
	2015	33, 26, 251	1, 0, 0	0, 0, 0	0, 0, 0	26, 6, 80	0, 0, 0	0, 3, 1	

*Abbreviations*: *A* adults, *L* larvae, *N* nymphs

**Table 2:** Average and peak density of *I. pacificus* adults, nymphs and larvae in 2013–2014 and 2014–2015 by reserve (site). Densities are presented as number of ticks per 100 m^2^ with standard errors in parentheses

		Average density		Peak density	
Life stage	Site	2013–2014	2014–2015	2013–2014	2014–2015
Adults	COPR	0.017 (0.009)	0.005 (0.003)	0.1 (0.045)	0.06 (0.031)
	Paradise	0.568 (0.147)	0.615 (0.164)	1.25 (0.210)	1.3 (0.520)
	Sedgwick	0.039 (0.009)	0.027 (0.011)	0.28 (0.074)	0.208 (0.060)
Adults total		0.118 (0.047)	0.115 (0.053)	0.367 (0.096)	0.328 (0.122)
Nymphs	COPR	0 (0)	0 (0)	0 (0)	0 (0)
	Paradise	0.109 (0.050)	0.007 (0.007)	0.359 (0.123)	0.036 (0.036)
	Sedgwick	0.007 (0.004)	0.025 (0.018)	0.06 (0.031)	0.147 (0.102)
Nymphs total		0.021 (0.011)	0.012 (0.008)	0.085 (0.034)	0.067 (0.044)
Larvae	COPR	0 (0)	0 (0)	0 (0)	0 (0)
	Paradise	0.297 (0.175)	0.201 (0.150)	0.959 (0.389)	0.6 (0.356)
	Sedgwick	0.024 (0.024)	0.275 (0.204)	0.38 (0.38)	1.953 (1.311)
Larvae total		0.06 (0.036)	0.148 (0.089)	0.318 (0.178)	0.914 (0.564)

**Fig. 2:**
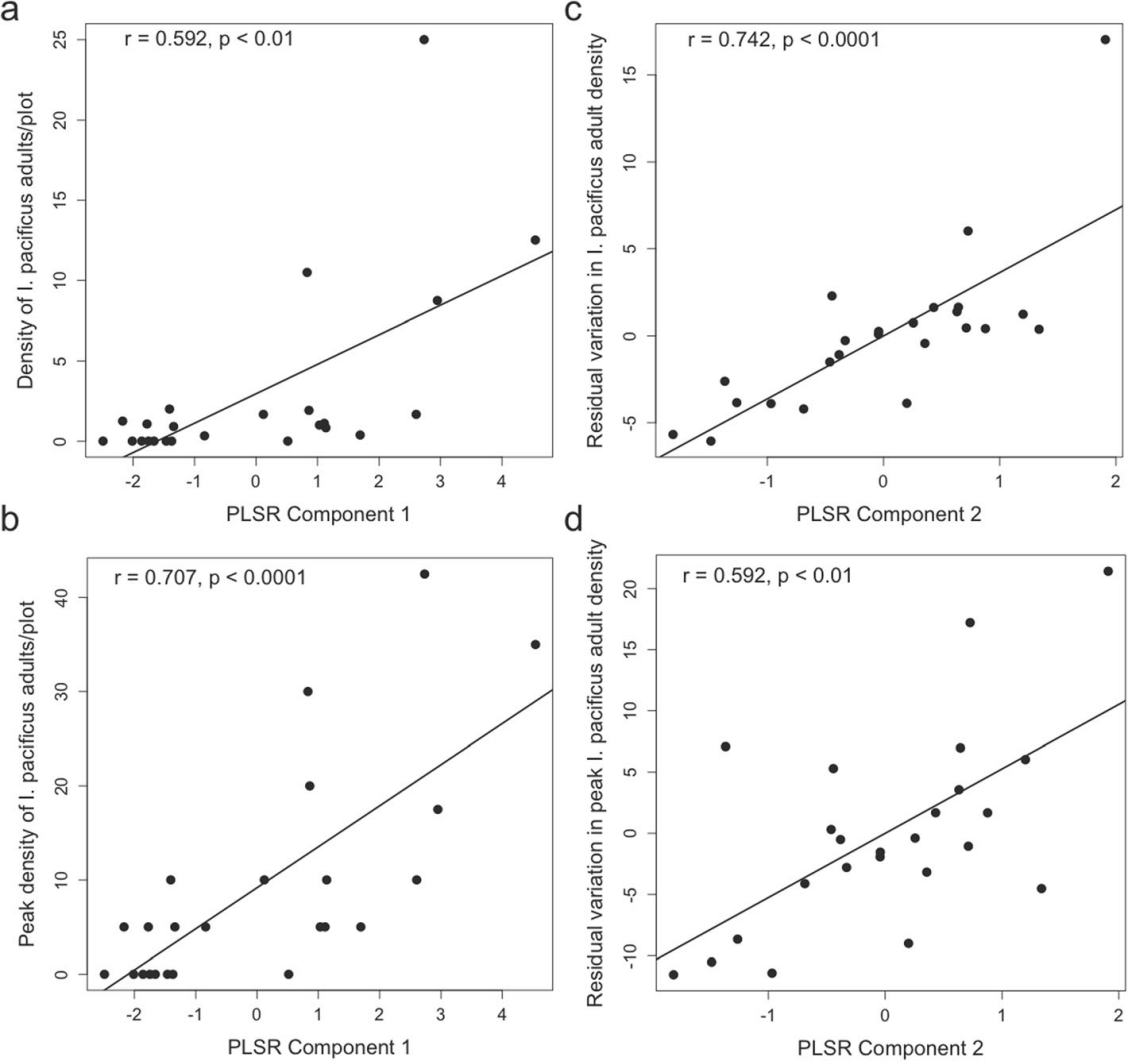
Density (**a**) and peak density (**b**) of adult *I. pacificus* in 2013–2014 plotted against the position of each sampled plot in the first PLSR component; and residual variation in density (**c**) and residual variation in peak density (**d**) plotted against the position of each sampled plot in the second PLSR component. Correlation coefficients and *P*-values are presented in each panel

In the first year of the study (2013–2014), adult *I. pacificus* density was primarily explained by woodrat nest density and dense oak woodland habitat. Two significant components were extracted in the multivariate model that explained 71.8% of the original variance in the response variables (Table 3). The first component accounts for a reasonably large proportion of the overall variance (~45%), while the second component accounts for a smaller, but significant proportion (~27%). The underlying drivers of these two components can be deduced from the variable weights and variable importance in the projection (VIP) scores (Table 3). The sum of squared variable weights for each component is equal to one, so the relative contribution of individual variables to the meaning of each component, and thus to the interpretation of the factors driving tick density, can be estimated [27]. In the multivariate model that predicts average and peak density of adult *I. pacificus* in 2013–2014, the first component is primarily determined by woodrat nest density (~43%), with stem density, canopy cover and elevation all contributing > 10% of the information content of the first component (Table 3). These four variables are not independent, but rather define an environment/habitat type that can be characterized as dense oak woodland. The significance of woodrat nest density here also suggests that host availability is an important driver of this first component. The VIP scores, which are calculated as the weighted sum of squares of the PLS weights, taking into account the explained variance of each PLS component, confirm that these same four variables (woodrat density, stem density, canopy cover and elevation) drive the relationship between the first component and adult tick density. The correlation between average and peak adult tick density in 2013–2014 and the position of the 24 plots in the first component of this PLSR model are shown in Fig. 2a, b (*r* = 0.592, *P* < 0.01; and *r* = 0.707, *P* < 0.0001, respectively). The second component is applied to the residual variation not explained by the first component, illustrated in Fig. 2c, d (*r* = 0.742, *P* < 0.0001; and *r* = 0.592, *P* < 0.01, respectively). Variable weights and VIP scores indicate that the residual variation in adult tick density is primarily explained by woodrat density, as well as a negative association with slope, canopy cover and stem density (Table 3). This set of explanatory variables suggests an association with both forested and more open grassland/chaparral habitats, which may be indicative of an association with the forest edge ecotone. Additionally, the significance of woodrat nest density may indicate the importance of juvenile tick host availability in predicting adult tick density.

**Table 3:** PLSR Model results for multivariate adult *I. pacificus* average and peak density in 2013–2014 and 2014–2015. VIP scores greater than 1 (highlighted in bold) indicate significant contributions of those variables to the variation explained by each component; variable weights indicate the direction of the effect. Asterisk indicates the variable contributing most significantly to the variation in the component, and the strongest predictor of tick density. The second component acts on residual variation not explained by the first component

	2013–2014		2014–2015	
	Weights (VIP) comp. 1	Weights (VIP) comp. 2	Weights (VIP) comp. 1	Weights (VIP) comp. 2
Avg. max. winter temp. 2013–2014	-0.313 (0.990)	-0.152 (0.837)	**-0.298 (1.033)**	0.015 (0.806)
Elevation (m)	**0.337 (1.067)**	-0.136 (0.885)	0.272 (0.944)	-0.089 (0.761)
Slope	0.144 (0.454)	**-0.490 (1.012)**	0.109 (0.379)	**-0.499 (1.122)**
Canopy cover (%)	**0.355 (1.122)**	**-0.298 (1.058)**	**0.298 (1.032)**	**-0.306 (1.043)**
Litter cover (%)	0.246 (0.777)	-0.389 (0.972)	0.229 (0.792)	-0.330 (0.946)
Shrub cover (%)	0.021 (0.067)	0.077 (0.158)	0.041 (0.143)	0.066 (0.181)
Grass cover (%)	-0.136 (0.431)	0.092 (0.384)	-0.147 (0.508)	0.063 (0.419)
Bare ground cover (%)	0.023 (0.071)	0.082 (0.167)	0.021 (0.074)	0.146 (0.321)
Stem density (No./Plot)	**0.367 (1.160)**	**-0.310 (1.097)**	**0.351 (1.216)**	**-0.255 (1.098)**
Woodrat density	0.654 (2.067)*	0.601 (2.007)*	0.631 (2.187)*	0.615 (2.165)*
Avg. max. winter temp. 2014–2015	na	na	**-0.350 (1.211)**	0.036 (0.947)
Avg. max. summer temp. 2014	na	na	-0.128 (0.444)	0.257 (0.656)
*R*-squared	0.45	0.268	0.442	0.285

*Abbreviations*: *na* not available, *VIP* variable importance in the projection

Similarly, in the second year of the study adult *I. pacificus* density was again explained by woodrat nest density and dense oak woodland habitat. The multivariate model of average and peak adult tick density the following year, in 2014–2015, yielded 2 significant components explaining 72.8% of the variance in the outcome variables (~44 and ~29% for component 1 and 2, respectively). VIP scores indicate that woodrat density, stem density and a negative association with average daily maximum rainy season temperature in the year of tick collection are driving the relationship between the first component and adult tick density in 2014–2015 (Table 3). Again, the residual variation is primarily explained by woodrat density, as well as a negative association with slope, stem density and canopy cover (Table 3). The multivariate PLSR models of average and peak adult *I. pacificus* density specified for each year of the study thus yielded remarkably consistent results.

**Fig. 3:**
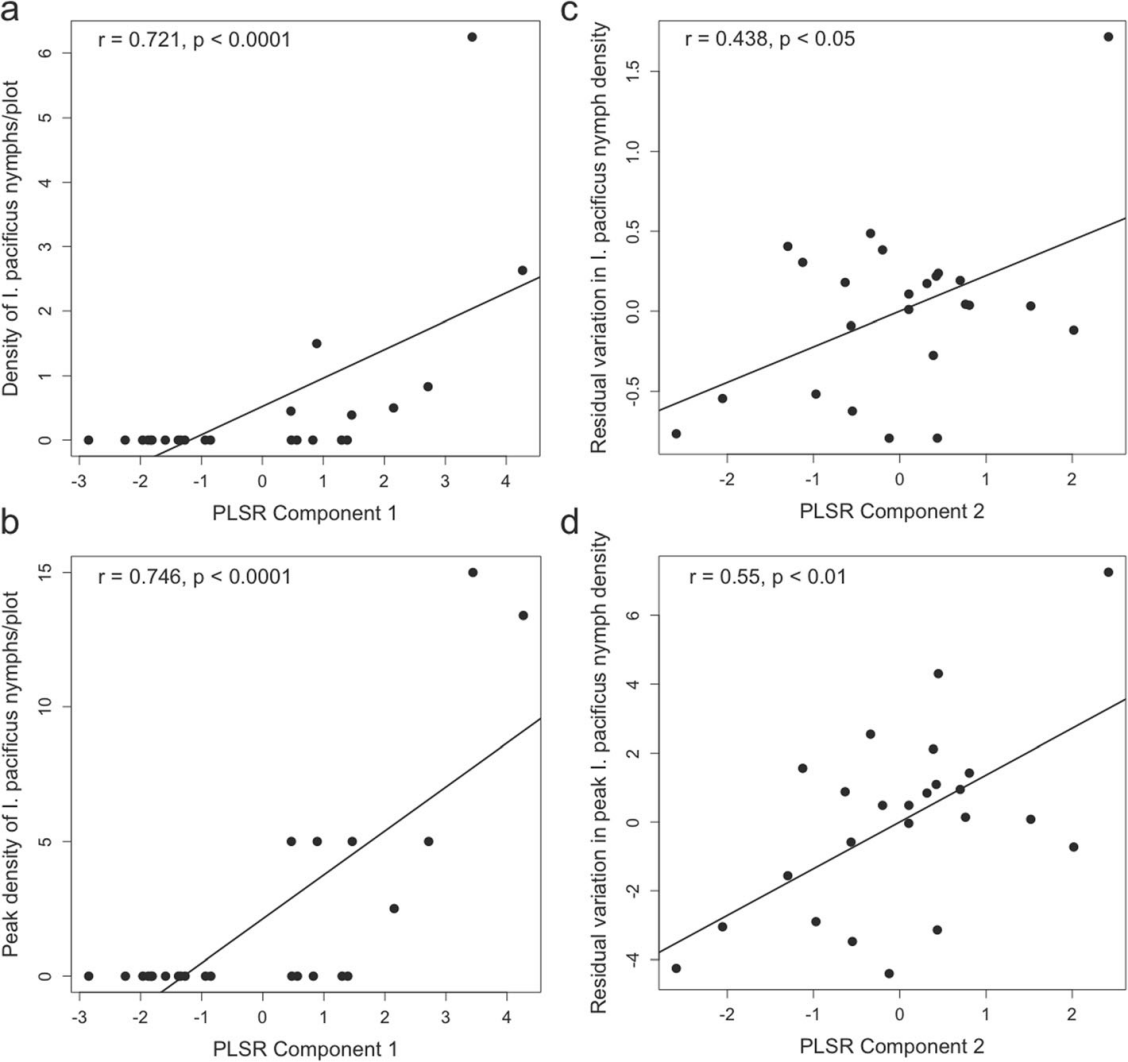
Density (**a**) and peak density (**b**) of nymphal *I. pacificus* in 2013–2014 plotted against the position of each sampled plot in the first PLSR component; and residual variation in density (**c**) and residual variation in peak density (**d**) plotted against the position of each sampled plot in the second PLSR component. Correlation coefficients and *P*-values are presented in each panel

The variables that explained patterns of nymphal tick density in 2013–2014 were similar to those of adult ticks, with woodrat density and dense oak woodland habitat driving the relationship. However, woodrat density was no longer a significant predictor in the second year of study, with variables associated with dense oak woodland driving the relationship. Results of the multivariate models of average and peak nymphal *I. pacificus* density were not as strong as those predicting adult tick density and not as consistent between the 2 years of the study. In 2013–2014, 2 extracted components explained 66% of the variation in the outcome variables (~48 and ~18% by component 1 and 2, respectively). VIP scores indicate that woodrat density, stem density and canopy cover are driving the relationship between the first component and nymphal tick density (Table 4; Fig. 3). The residual variation is primarily explained by woodrat nest density, bare ground cover, as well as negative associations with stem density, slope and canopy cover. In the second year of the study, 2 extracted components explained only 54% of the variation in average and peak nymphal tick density (~30 and ~25% by component 1 and 2, respectively). The VIP scores indicate that woodrat density was no longer a significant predictor in 2014–2015, instead percent leaf litter cover was the strongest predictor with stem density, canopy cover, elevation and a negative association with grass/herbaceous cover also contributing to the relationship between the first component and nymphal tick density (Table 4). Residual variation was explained primarily by leaf litter cover, as well as negative associations with woodrat density, stem density and canopy cover (Table 4).

**Table 4:** PLSR Model results for multivariate nymphal *I. pacificus* average and peak density in 2013–2014 and 2014–2015. VIP scores greater than 1 (highlighted in bold) indicate significant contributions of those variables to the variation explained by each component; variable weights indicate the direction of the effect. Asterisk indicates the variable contributing most significantly to the variation in the component, and the strongest predictor of tick density. The second component acts on residual variation not explained by the first component

	2013–2014		2014–2015	
	Weights (VIP) comp. 1	Weights (VIP) comp. 2	Weights (VIP) comp. 1	Weights (VIP) comp. 2
Avg. max. winter temp. 2013–2014	-0.187 (0.590)	0.081 (0.522)	0.021 (0.073)	0.377 (0.882)
Elevation (m)	0.312 (0.986)	-0.237 (0.929)	**0.313 (1.083)**	-0.162 (0.885)
Slope	0.071 (0.223)	**-0.637 (1.059)**	0.228 (0.789)	-0.238 (0.806)
Canopy cover (%)	**0.386 (1.220)**	**-0.225 (1.107)**	**0.307 (1.063)**	**-0.308 (1.065)**
Litter cover (%)	0.299 (0.946)	-0.278 (0.929)	0.706 (2.445)*	0.403 (2.036)*
Shrub cover (%)	0.055 (0.173)	0.116 (0.241)	0.007 (0.026)	0.005 (0.023)
Grass cover (%)	-0.235 (0.742)	-0.082 (0.649)	**-0.317 (1.098)**	-0.184 (0.918)
Bare ground cover (%)	0.249 (0.788)	**0.534 (1.104)**	-0.068 (0.235)	-0.025 (0.183)
Stem density (No./Plot)	**0.470 (1.485)**	**-0.043 (1.273)**	**0.365 (1.266)**	**-0.234 (1.082)**
Woodrat density	0.533 (1.686)*	0.310 (1.529)*	0.121 (0.419)	**-0.413 (1.013)**
Avg. max. winter temp. 2014–2015	na	na	-0.063 (0.218)	0.372 (0.885)
Avg. max. summer temp. 2014	na	na	0.017 (0.059)	0.346 (0.809)
*R*-squared	0.484	0.177	0.295	0.247

*Abbreviations*: *na* not available, *VIP* variable importance in the projection

**Fig. 4:**
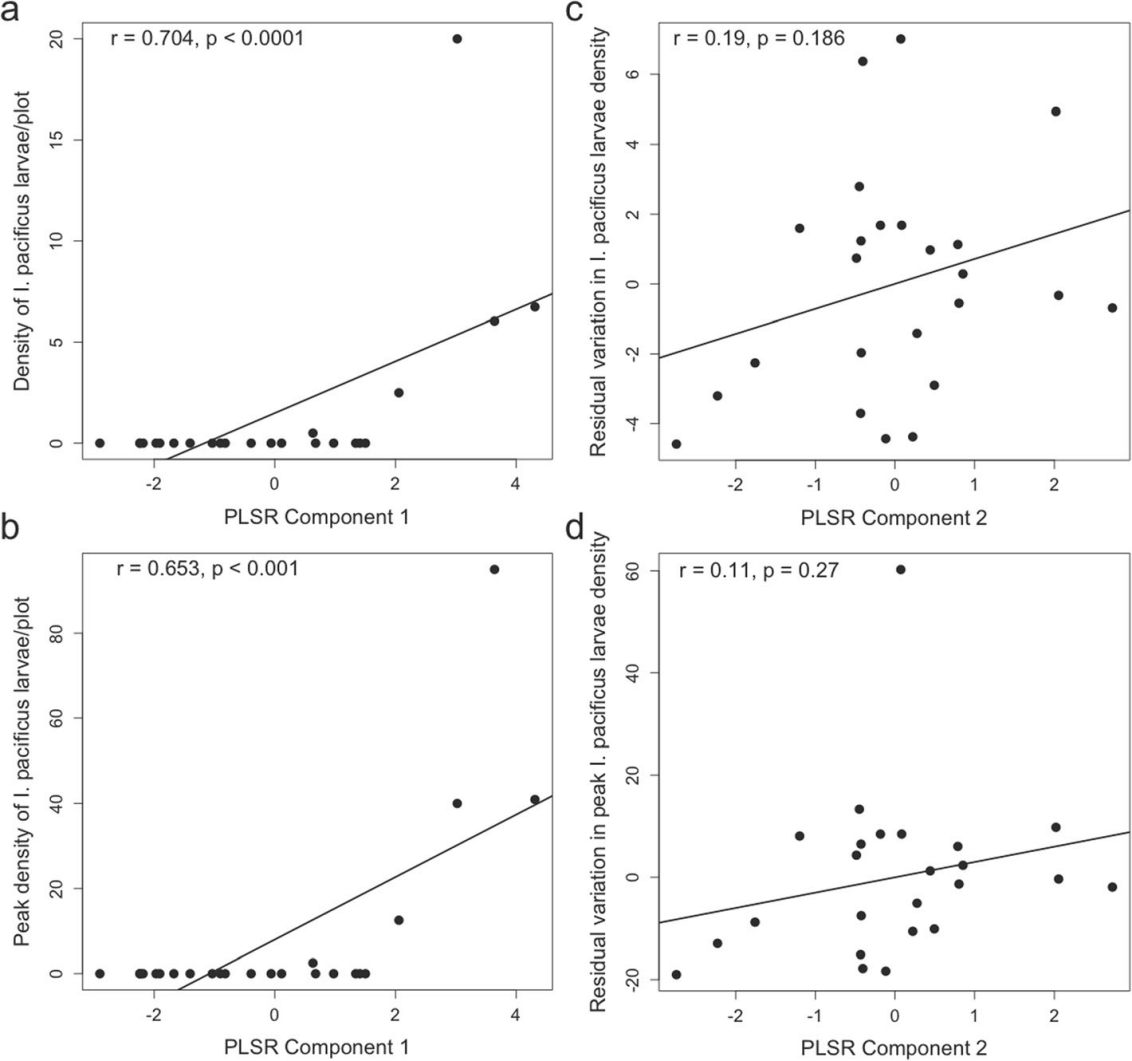
Density (**a**) and peak density (**b**) of larval *I. pacificus* in 2013–2014 plotted against the position of each sampled plot in the first PLSR component; and residual variation in density (**c**) and residual variation in peak density (**d**) plotted against the position of each sampled plot in the second PLSR component. Correlation coefficients and *P*-values are presented in each panel

The models of larval *I. pacificus* density show remarkably similar patterns to models of nymphal ticks, suggesting immature stages responded similarly to abiotic conditions in this study. In 2013–2014, 2 extracted components explained 48% of the variation in the outcome variables (~40 and ~8% by component 1 and 2, respectively). VIP scores indicate that woodrat density, stem density, canopy cover, leaf litter cover, as well as negative associations with grass cover are driving the relationship between the first component and larval tick density (Table 5; Fig. 4). The residual variation is primarily explained by woodrat nest density, as well as negative associations with leaf litter cover, canopy cover, grass cover and stem density. In the second year of the study, 2 extracted components explained 63% of the variation in larval tick density (~36 and ~27% by component 1 and 2, respectively). The VIP scores again indicate that woodrat density was no longer a significant predictor in 2014–2015, instead percent leaf litter cover was the strongest predictor with stem density, canopy cover, elevation and a negative association with grass/herbaceous cover also contributing to the relationship between the first component and larval tick density (Table 5). Residual variation was explained primarily by leaf litter cover, as well as negative associations with stem density, grass cover and canopy cover (Table 5).

**Table 5:** PLSR Model results for multivariate larval *I. pacificus* average and peak density in 2013–2014 and 2014–2015. VIP scores greater than 1 (highlighted in bold) indicate significant contributions of those variables to the variation explained by each component; variable weights indicate the direction of the effect. Asterisk indicates the variable contributing most significantly to the variation in the component, and the strongest predictor of tick density. The second component acts on residual variation not explained by the first component

	2013–2014		2014–2015	
	Weights (VIP) comp. 1	Weights (VIP) comp. 2	Weights (VIP) comp. 1	Weights (VIP) comp. 2
Avg. max. winter temp. 2013–2014	-0.178 (0.561)	0.112 (0.533)	-0.017 (0.057)	0.395 (0.895)
Elevation (m)	0.281 (0.890)	-0.258 (0.878)	**0.304 (1.054)**	-0.123 (0.849)
Slope	0.124 (0.391)	-0.647 (0.901)	0.214 (0.741)	-0.254 (0.804)
Canopy cover (%)	**0.351 (1.111)**	**-0.281 (1.078)**	**0.311 (1.078)**	**-0.293 (1.052)**
Litter cover (%)	**0.446 (1.411)**	**-0.048 (1.292)**	0.674 (2.335)*	0.443 (2.032)*
Shrub cover (%)	0.155 (0.491)	0.287 (0.580)	0.056 (0.194)	0.049 (0.184)
Grass cover (%)	**-0.356 (1.126)**	**-0.315 (1.106)**	**-0.343 (1.189)**	**-0.238 (1.049)**
Bare ground cover (%)	0.046 (0.145)	0.381 (0.504)	-0.088 (0.306)	-0.054 (0.261)
Stem density (No./Plot)	**0.438 (1.385)**	**-0.046 (1.268)**	**0.372 (1.289)**	**-0.215 (1.090)**
Woodrat density	0.455 (1.438)*	0.305 (1.371)*	0.176 (0.609)	-0.365 (0.946)
Avg. max. winter temp. 2014–2015	na	na	-0.101 (0.351)	0.377 (0.895)
Avg. max. summer temp. 2014	na	na	-0.043 (0.150)	0.312 (0.716)
*R*-squared	0.398	0.078	0.359	0.267

*Abbreviations*: *na* not available, *VIP* variable importance in the projection

### Drivers of infection prevalence

Infection with *Borrelia burgdorferi* (*s.l*.) was extremely uncommon throughout the study area (Table 6). No *I. pacificus*, the primary vector of *B. burgdorferi* (*s.s*.) to humans in the western US, were infected with any spirochete in the *B. burgdorferi* (*s.l*.) complex out of 288 adults and 67 nymphs that were tested in this study. However, there was evidence that other *Ixodes* spp. ticks in this region may be positive for infection with *B. burgdorferi* (*s.s*.), the causative agent of Lyme disease, as well as with *Borrelia bissettiae*, another spirochete in the *B. burgdorferi* (*s.l*.) complex that may be involved in Lyme borreliosis in central and southern Europe [30–32]. *Borrelia bissettiae* has also been isolated from ticks and hosts throughout the US [33], and DNA resembling *B. bissettiae* has been isolated from the serum of a Lyme disease patient in northern California [34], though illness associated with *B. bissettiae* in North America is not well established. Specifically, in this study three of six (50%) *Ixodes peromysci* tested were positive for *B. burgdorferi* (*s.l*.), all from Coal Oil Point Reserve (Fig. 1). *Ixodes peromysci* is a specialist on deer mice (*Peromyscus maniculatus*) and other small rodents [35]. In this study, one nymph was infected with *B. burgdorferi* (*s.s*.) and two nymphs were infected with *B. bissettiae* (Table 6). In addition, one out of nine (11%) *Ixodes spinipalpis* tested was positive for *B. burgdorferi* (*s.l*.), again from Coal Oil Point Reserve. *Ixodes spinipalpis* is thought to play a role in the enzootic maintenance of *B. burgdorferi* (*s.l*.) in California [24, 25] and one adult female was found to be infected with *B. bissettiae* in this study (Table 6). However, both of these species of tick very rarely bite humans, so present very little risk of human infection.

**Table 6:** Summary of infection results by tick species

Tick species	Number tested	Number infected	*B. burgdorferi* (*s.l*.) type	Prevalence (%) (all life stages)
*I. pacificus*	288 adults; 67 nymphs	0	na	na
*I. brunneus*	2 adults; 4 nymphs	0	na	na
*I. spinipalpis*	1 adult; 8 nymphs	1 adult female	*B. bissettiae*	11.1
*I. peromysci*	1 adult; 5 nymphs	3 nymphs	*B. burgdorferi* (*s.s*.) (1); *B. bissettiae* (2)	50.0

*Abbreviation*: *na* not available

Due to the lack of infection in ticks in 2013–2014, PLSR models predicting infection were performed only for the 2014–2015 season and were not run for *I. pacificus*, which were not infected in this study. In addition to the environmental and habitat variables used in the models that predicted tick density, tick species diversity (Shannon’s H) was included in the set of predictors for the model of infection prevalence (Tables 1 and 7). The two extracted components explained 62% of the variation in infection prevalence (~53 and ~9% by component 1 and 2, respectively). In contrast to models that predicted density of *I. pacificus*, VIP scores indicate that infection prevalence in the tick community was most strongly influenced by vector diversity and both summer and winter temperature, rather than by woodrat density or dense oak woodland. There was a strong positive association between tick diversity and infection prevalence with *B. burgdorferi* (*s.l*.) spirochetes in the tick community (Table 7). In addition, there was a strong negative association with maximum summer temperature, and positive association with maximum winter temperature (Table 7).

**Table 7:** PLSR Model results for infection prevalence with *Borrelia burgdorferi* (*s.l*.) in *Ixodes* spp. ticks, 2014–2015. VIP scores greater than 1 (highlighted in bold) indicate significant contributions of those variables to the variation explained by each component; variable weights indicate the direction of the effect. Asterisk indicates the variable(s) contributing most significantly to the variation in the component, and the strongest predictor of infection. The second component acts on residual variation not explained by the first component

	Weights (VIP) comp. 1	Weights (VIP) comp. 2
Avg. max. winter temp. 2013–2014	-0.040 (0.144)	0.442 (1.594)*
Elevation (m)	**-0.278 (1.001)**	-0.025 (0.092)
Slope	-0.103 (0.373)	-0.186 (0.670)
Canopy cover (%)	-0.091 (0.328)	-0.239 (0.861)
Litter cover (%)	0.123 (0.443)	-0.211 (0.761)
Shrub cover (%)	0.156 (0.561)	**-0.373 (1.344)**
Grass cover (%)	-0.144 (0.518)	**0.381 (1.375)**
Bare ground cover (%)	**-0.296 (1.068)**	**0.311 (1.120)**
Stem density (No./Plot)	-0.065 (0.234)	-0.202 (0.729)
Woodrat density	-0.144 (0.518)	-0.185 (0.667)
Tick diversity (Shannon’s)	0.603 (2.174)*	0.224 (0.807)
Avg. max. winter temp. 2014–2015	0.012 (0.044)	**0.401 (1.446)**
Avg. max. summer temp. 2014	-0.605 (2.181)*	-0.034 (0.124)
*R*-squared	0.529	0.086

*Abbreviation*: *VIP* variable importance in the projection

## Discussion

In order to better understand and manage risk of vector-borne and zoonotic disease an understanding of the environmental conditions that promote vector populations and infection prevalence with zoonotic pathogens on the landscape is increasingly necessary [2, 5–7, 36]. In northwestern California, the region of western North America with the highest human incidence of Lyme disease [37], the abiotic, habitat and environmental conditions that might promote vector density and infection prevalence have been investigated in numerous studies [10, 11, 13–15, 38–40]. While there is substantial natural heterogeneity in density and infection prevalence of *I. pacificus* in California, both over space and through time [9, 15], these previous studies have largely identified dense oak woodlands with microclimates that maintain high relative humidity and small temperature fluctuations, particularly in the summer months, as high-risk areas for Lyme disease [10, 11, 13–15, 38–40]. In southern California, one of the most densely populated regions of the US, Lyme disease ecology and environmental risk factors have not been well explored, though there is evidence to suggest the ecology and epidemiology of the disease may differ from northwestern California [16, 17].

In this study, density of *I. pacificus* ticks across all life stages, particularly the nymphal stage, and across all plots and years sampled was very low compared to previous density estimates from similar studies conducted in northwestern California [15, 41]. For example, previous estimates of peak adult density in northern California range from ~15 to ~380 ticks per 100 m^2^ [41] compared to < 1 in the present study to ~3 ticks per 100 m^2^ in a recent study conducted in southern California [16], with similar patterns observed for nymphal and larval ticks. This may be due in part to the worsening drought conditions experienced in California over the course of the present study, which would obscure direct comparison with earlier studies conducted in northern California in the absence of baseline data from southern California. However, this may also suggest actual differences in baseline density of *I. pacificus* between these two regions. For example, a previous study [17] found nymphal density to be extremely low in southern California in years prior to the recent drought. Furthermore, interannual patterns of nymphal and adult tick density in southern California suggest a significant difference in questing behavior of this life stage between northwestern and southern California.

Adult tick density in year *t* should be determined by nymphal tick density in year *t* – 1, yet questing nymphal tick density in year *t* – 1 is substantially lower than adult tick density in year *t* in both the present study as well as in a recent study conducted in southern California [16]. This hourglass-shaped demographic distribution, with much higher densities of adult and larval ticks than nymphal ticks, contrasts with the expected pyramid-shaped distribution found in other studies in northern California [41]. This curious demographic pattern suggests that nymphal ticks are not being captured at the same rate by the flagging method in southern California as they are in northern California, which may indicate a regional difference in questing behavior of this stage similar to patterns observed in the southeastern US with nymphal *I. scapularis* [42]. In the northeastern US all life stages of *I. scapularis* are readily collected by flagging, while in the southeast the same method collects very few juvenile stages, even at sites where adults are readily collected [42]. A recent study investigated whether this may be due to differences in nymphal *I. scapularis* questing behavior between northern and southern populations, and found strong evidence suggesting that nymphal *I. scapularis* from southern populations rarely emerge from leaf litter to quest for hosts, while nymphs from northern populations do [42]. This behavioral difference has significant implications for disease transmission to humans [42], and may also be characteristic of populations of *I. pacificus* in western North America.

In this study, the abiotic and environmental drivers of variation in tick density and infection prevalence in southern California were investigated using a partial least squares regression approach. Results from models predicting adult *I. pacificus* density were consistent across years and suggest that host availability for juvenile ticks was the most significant factor predicting adult tick density (i.e. VIP scores were substantially higher for woodrat density in both extracted components than for other significant predictors). Dense oak woodland and concurrent average daily maximum rainy season temperature were also found to be important drivers of adult tick density, as predicted, though not as strong as host availability for juvenile *I. pacificus*. While the possibility exists that oak woodland habitat may be a driver of both tick and woodrat abundance, particularly in some regions of California, in this study woodrats were abundant in both chaparral- and oak woodland-dominated plots suggesting that the relationship between woodrat abundance and tick abundance is likely not due to a spurious correlation. The significance of rainy season temperature, concurrent with tick questing, for adult *I. pacificus* density suggests that high rainy season temperatures may inhibit adult tick questing activity. Rainy season temperatures may be as significant as summer dry season conditions and average maximum daily temperature in the previous summer [13, 15] in determining the likelihood of encountering adult *I. pacificus* ticks in southern California, and suggests that winter temperatures may also be an important limitation on adult tick populations.

Results from models predicting both nymphal and larval *I. pacificus* density were less consistent between years, and explained less of the variation in tick density than did models for adult tick density. This may have been due to sampling bias for these life stages, since comparatively few larval and nymphal ticks were collected in this study, as in previous studies conducted in southern California [16, 17]. Additionally, variables that were not measured in this study, such as the density of western fence lizards, may be relatively more important for juvenile ticks than adult ticks in determining abundance. Omission of such predictor variables could weaken inference on the included predictor variables. In the first year of the study, results suggest that host availability for juvenile ticks was the strongest predictor of both nymphal and larval density, with variables characteristic of dense oak woodland also found to be significant predictors. However, in the second year of the study leaf litter cover was the strongest predictor of both nymphal and larval density, and host availability for juvenile ticks was no longer significant. This may suggest that as drought conditions worsened in California from 2013 through 2015, habitat characteristics - like dense leaf litter and overstory canopy - that promote the microclimatic conditions necessary for tick survivorship (i.e. lower temperatures and higher relative humidity) became more important than host availability as predictors of larval and nymphal tick density.

The finding that no *I. pacificus* ticks were infected with *B. burgdorferi* (*s.l*.) spirochetes in this study suggests that human risk of Lyme disease in this region is exceedingly low. However, the identification of infected *I. spinipalpis* and *I. peromysci* ticks, both of which very rarely bite humans, is significant. This finding suggests that despite possible enzootic transmission of the pathogen, populations of *I. pacificus* in southern California are not interacting with enzootic transmission cycles, leading to low human risk. Perhaps this lack of infection in *I. pacificus* populations is the result of low rates of blood feeding on reservoir hosts relative to non-competent hosts like western fence lizards (*S. occidentalis*). It may also be due to small sample sizes, particularly of the nymphal stage (*n* = 67), which would make identification of infected *I. pacificus* unlikely if rates of infection are low. The exact mechanism behind this pattern of infection warrants further investigation, but suggests that pathogen transmission to humans through the bite of infected *I. pacificus* ticks in this region is highly unlikely. This pattern of infection may also suggest that enzootic transmission of *B. burgdorferi* (*s.l*.) in natural transmission cycles involving alternative vector species, other than *I. pacificus*, and reservoir hosts may be occurring more widely in California than studies focused on the northwestern region of the state might suggest. This may be particularly relevant in the comparatively understudied central and south coastal regions of the state. However, these particular tick species and associations with *B. burgdorferi* (*s.l*.) genospecies require further investigation, including transmission experiments, to determine whether these results are truly indicative of novel enzootic transmission cycles.

Results also suggest that diversity in the vector community may predict infection prevalence in tick populations, and perhaps in the host community as a result. In this study, low diversity vector communities were comprised of common generalist species like *I. pacificus* and *D. occidentalis*, while higher diversity communities included both common generalist species as well as less common specialists like *I. brunneus*, *I. spinipalpis* and *I. peromysci*. The higher diversity communities thus included alternative vector species thought to contribute to enzootic maintenance of *B. burgdorferi* (*s.l*.), explaining the relationship between vector diversity and infection prevalence in this region and this particular study. The identity of the tick species present in each community sampled was thus critically important in determining the presence of *B. burgdorferi* (*s.l*.), suggesting that tick species identity rather than species diversity, *per se*, may be the key factor associated with infection prevalence in this region of California. Additionally, due to the lack of explanatory power of dusky-footed woodrat density for infection prevalence in ticks, this suggests that alternative reservoir hosts, like western gray squirrels or deer mice, may be playing a more important role in the enzootic transmission of tick-borne borreliae in southern California. The significant negative relationship between maximum summer temperature and infection prevalence further corroborates the results of earlier studies suggesting that microclimates characterized by high relative humidity and small temperature fluctuations are more likely to harbor infected ticks in California. This also suggests that the protracted drought in California is negatively impacting tick populations and disease risk. However, due to the comparative rarity of infection in the vector community in this study, it is possible that unmeasured explanatory factors (e.g. host community composition) may be important in determining infection prevalence or whether these pathogens have arrived or could be maintained in particular sites where they were found to be absent.

The results of this study, in addition to identifying possible abiotic and environmental risk factors for Lyme disease in southern California, may have implications for tick-borne disease risk in the western US under climate change. Evidence suggests that the geographic range of the vector in the eastern US, *I. scapularis*, is increasing as a result of climate change, driving increases in regional Lyme disease risk [43–47]. This is likely due to increases in the basic reproductive number, R_0_, of the vector, resulting from increased molting success and survivorship and accelerated phenology of *I. scapularis* due to milder winters [46]. However, it is currently unknown whether Lyme disease foci in the western US will experience similar range shifts and changes in Lyme disease risk due to climate change. While growth and survival of *I. scapularis* appears to be limited primarily by winter conditions, *I. pacificus*, the primary Lyme disease vector in the western US, in contrast is largely limited by abiotic conditions in the summer dry season [10, 13, 15]. The results of this study further substantiate the importance of habitat and abiotic conditions that create microclimates that protect desiccation prone ticks from high temperatures and low relative humidity, as well as promote the necessary host populations for maintenance of *B. burgdorferi* (*s.l*.) in enzootic transmission cycles. Moreover, in this study it appears that these factors were increasingly important as California’s historic drought progressed. In California, temperatures are expected to increase, and precipitation decrease into the future, particularly in northern coastal California [48, 49], exacerbating seasonal drought. We hypothesize that these expected climate change impacts may then serve to limit *I. pacificus* populations, leading to reduced human risk in California, though may also serve to increase risk in the northwestern US on the northern edge of the Lyme disease foci in western North America. However, the 2 years of data in this study are not sufficient to draw concrete conclusions, and further study of this relationship is necessary to identify the direct effect of climate change on tick populations. Furthermore, the response of the pathogen to climate change, through altered replication rates or interactions between reservoir hosts and competent vectors, also remains uncertain.

## Conclusions

In this study, dense oak woodland habitat and abundant small vertebrate hosts were found to be the strongest predictors of *I. pacificus* tick abundance, particularly the nymphal stage. However, overall density of *I. pacificus* was found to be quite low in comparison to well-studied sites in northwestern California [15, 41]. Furthermore, habitat types and microclimatic conditions thought to buffer juvenile ticks from desiccation over the summer dry season were found to be increasingly important as the study, and California’s historic drought, progressed. These results suggest that drought in California may act as an important limiting factor on *I. pacificus* populations though further investigation is necessary. Additionally, infection prevalence with *B. burgdorferi* (*s.l*.) was exceedingly low or zero across all plots sampled in this study, in contrast with northwestern California. However, despite the low risk of human Lyme disease infection posed by *I. pacificus* in southern California, comparatively high rates of infection were detected in other *Ixodes* spp. ticks. While *I. spinipalpis* and *I. peromysci* may be playing a role in enzootic transmission of *B. burgdorferi* (*s.l*.) in southern California, these species do not commonly bite humans and thus pose little risk for human infection. However, the roles of these tick species in enzootic transmission cycles, and whether *I. pacificus* might be involved, require further investigation. Finally, infection prevalence was not associated with habitats characterized by oak woodland nor with the presence of dusky-footed woodrats in this study, which suggests that different vector species and reservoir hosts may be playing a key role in the ecology of the transmission cycle in this region.

## Abbreviations

PLSR: Partial least squares regression
R_0_: Basic reproductive number
UCNRS: University of California Natural Reserve System
VBZD: Vector-borne zoonotic disease
VIP: Variable importance in the projection scores, calculated in PLSR
